# Efficacy of Two Commercial Ready-To-Use PCV2 and *Mycoplasma hyopneumoniae* Vaccines under Field Conditions

**DOI:** 10.3390/ani11061553

**Published:** 2021-05-26

**Authors:** Gonzalo López-Lorenzo, Alberto Prieto, Cynthia López-Novo, Pablo Díaz, Ceferino Manuel López, Patrocinio Morrondo, Gonzalo Fernández, José Manuel Díaz-Cao

**Affiliations:** INVESAGA Group, Department of Animal Pathology, Faculty of Veterinary Sciences, Universidade de Santiago de Compostela, Avd. Carballo Calero s/n, 27002 Lugo, Spain; gonzalo.lopez.lorenzo@gmail.com (G.L.-L.); alberto.prieto@usc.es (A.P.); pablo.diaz@usc.es (P.D.); c.lopez@usc.es (C.M.L.); patrocinio.morrondo@usc.es (P.M.); gonzalo.fernandez@usc.es (G.F.); jmdchh@gmail.com (J.M.D.-C.)

**Keywords:** PCV2, *Mycoplasma hyopneumoniae*, ready-to-use, vaccination, sow parity, field trial

## Abstract

**Simple Summary:**

Porcine Circovirus Type 2 (PCV2) and *Mycoplasma hyopneumoniae* are widespread pathogens which cause a negative health impact on swine and thus lead to important economic losses in farms. Vaccination is the main preventive measure for both infections and, since the recommended vaccination programs against both pathogens are very similar, bivalent vaccines are commercially available. However, the immune status of the sows against these infections could interfere in the efficacy of these products. The aim of this study was to assess the efficacy of two ready-to-use vaccines against PCV2 and *M. hyopneumoniae* in a herd positive to both infections under field conditions, considering the sow parity of the studied piglets. Both vaccines were efficient in preventing the development of PCV2 viremia, *M. hyopneumoniae* lung lesions and improving pig growth. Nevertheless, only one of them showed a significant improvement in the average daily weight gain and in the reduction of lung lesions when compared to the unvaccinated group. Sow parity did not interfere in the obtained outcomes. Considering the used vaccine, the anti-PCV2 antibody response was slightly different in pigs from primiparous sows than in those from sows with a higher parity number.

**Abstract:**

Porcine Circovirus Type 2 (PCV2) and *Mycoplasma hyopneumoniae* are economically important pathogens in swine farms. Vaccination is the main preventive measure for both infections. In order to test two ready-to-use bivalent vaccines, 646 piglets from a herd actively infected with both pathogens were stratified according to the sow parity number and randomly assigned to three groups: A and B were vaccinated with two different vaccines, respectively, while C remained as the unvaccinated control. Vaccine efficacy was assessed based on the weight, average daily weight gain (ADWG), degree of lung lesions, presence of PCV2 viremia by qPCR and presence of PCV2 and *M. hyopneumoniae* antibody levels by ELISA. Our data revealed that the sow parity did not influence the vaccine outcomes. Good results for most of the analyzed parameters were observed in both vaccinated groups. ADGW and final weight were higher and lung lesions were less evident in both vaccinated groups than in the control one, but only Group A showed a significant improvement. PCV2 viremia was not detected in Group A, but it did appear in Group B coinciding with its peak in Group C. Finally, both the PCV2 and *M. hyopneumoniae* serological patterns differed depending on the employed vaccine.

## 1. Introduction

Porcine Circovirus type 2 (PCV2) and *Mycoplasma hyopneumoniae* are two main pathogens of swine, present in most commercial farms [1,2]. Both infections produce a considerable health impact, affecting the whole fattening phase: while in PCV2 natural infections, the clinical signs are observed during the first few weeks of fattening [3], those due to *M. hyopneumoniae* occur towards the end of that production stage [4]. In addition, both infections contribute to the development of secondary infections; for example, bacteria such as *Actinobacillus pleuropneumoniae* are known to be able to proliferate as a consequence of *M. hyopneumoniae* infection [5]. As a consequence, a decrease in growth and an increase in the number of sick animals and in mortality rates can be observed [6,7].

Vaccination is a key measure to control these infections and its use is widely spread to prevent their associated losses [7,8]. Regarding PCV2, although the best results have been observed when pigs are vaccinated at 6 weeks old, the conventional PCV2 vaccination takes place at 3–4 weeks of age [9]. In the case of *M. hyopneumoniae*, piglets are thought to be firstly exposed to the pathogen during the lactation period; thus, the vaccination against this pathogen should also be performed in the first few weeks of life [7]. Therefore, applying vaccines that are simultaneously protective for both PCV2 and *M. hyopneumoniae* is epidemiologically justified by the following reasons: first, their efficacy is equivalent to that obtained when they are administered separately [10,11]; second, this strategy offers advantages to swine producers such as reducing the labor costs, the number of manipulations that the animals undergo and the risk of pathogen transmission through needles; third, this lower need for animal manipulation and injection leads to an improvement of piglet welfare.

A few ready-to-use vaccines are available in the market for both pathogens, but some important questions about their performance under field conditions still need to be addressed. For example, it has been reported that high levels of PCV2 maternal derived antibodies (MDA) interfere with the active humoral immune response following vaccination in piglets [12] and that the levels of PCV2 MDA in piglets may depend on the sow parity [13]. With respect to this, piglets with high PCV2 MDA in the moment of vaccination tend to show a lower average daily weight gain (ADWG) than those which get vaccinated when their MDA titers are low [14,15]. In the case of *M. hyopneumoniae*, piglets from primiparous sows have higher odds of being infected by *M. hyopneumoniae* earlier than those from multiparous sows [16]. The earlier colonization of these piglets determines the posterior clinical presentation and the disease severity at herd level, mainly because it increases the subsequent transmission between pen mates during the post-weaning and fattening periods [4,7]. Thus, the immune or microbiological status of the sows in the moment of vaccination may compromise the effectiveness of the vaccine against these pathogens. Considering that under field conditions it is difficult to have a completely homogeneous sow population, some effects of the ready-to-use vaccines may be interfered with by sow parity.

For the aforementioned reasons, the aims of this study were to compare, under field conditions, the improvement of production parameters and the virological, immunological and pathological outcomes of two groups of pigs vaccinated with different commercial ready-to-use PCV2 and *M. hyopneumoniae* vaccines regarding an unvaccinated group, as well as to investigate if the sow parity may influence the efficacy of these vaccines.

## 2. Materials and Methods

### 2.1. Herd Description and Previous Situation

This field study was conducted during the second half of 2019 in a commercial 250-sow Spanish farm managed in a 3-week batch system, in which the piglets were weaned at 28 days old. The farm was operated in a two-site production system and piglets were sent at the end of the nursery stage to a fattening pig unit located approximately 10 km away. The herd had a history of enzootic pneumonia and was endemically infected with Porcine Reproductive and Respiratory Syndrome (PRRS) virus (prior to the start of the trial, all piglets were vaccinated against PRRS and *M. hyopneumoniae* at 18 days old). In addition, vaccination against PCV2 was not routinely performed before the beginning of the study.

The presence of PCV2 and *M. hyopneumoniae* infections was confirmed prior to the start of the study. Regarding PCV2, an uneven growth was evident at 4 months old. Moreover, at that age, high viremia (from 3.79 × 10^4^ to 1.26 × 10^8^ PCV2 copies/mL serum) and high PCV2 amounts in dead pigs (up to 1.34 × 10^12^ PCV2 copies/g in samples from inguinal lymph nodes) were detected. Regarding *M. hyopneumoniae*, previous lung lesion evaluation showed an average of 3.10 out of 28 based on the Madec and Kobisch (1982) method [17]; additionally, PCR analysis confirmed the presence of this bacteria in the lung lesions and discarded other pathogens such as PRRS virus and Swine Influenza virus.

### 2.2. Study Design

All the protocols and procedures followed during the study were approved by the bioethics committee of the University of Santiago de Compostela (project identification code: 2019-CE194). The study included two consecutive batches. The parity of each sow (1st, 2nd to 4th and ≥5th) was identified, and cross-fostering, when needed, was only done within sows of the same parity. Vaccination against PRRS at 18 days old, which was routinely being performed in the farm, was administered to all the piglets included in this study. 

Three days before weaning (25 days old) each piglet was weighed and individually identified by an ear tag, excluding weak and unhealthy piglets. The following day (26 days old), the piglets were randomly distributed in three homogenous groups according to the piglet weight and the sow parity, and they were vaccinated in the following way:

- Group A: 213 piglets (58 from 1st parity sows, 111 from 2nd to 4th and 44 from ≥5th) vaccinated in the neck, by the intramuscular route, with 2 mL of Porcilis^®^ PCV M Hyo [18] (Intervet International B.V, Boxmeer, Netherlands).
- Group B: 207 piglets (57 from 1st parity sows, 108 from 2nd to 4th and 42 from ≥5th) vaccinated in the neck, by the intramuscular route, with 2 mL of Suvaxyn^®^ Circo+MH RTU [19] (Zoetis Belgium SA, Louvain-la-Neuve, Belgium).
- Group C: 226 piglets (59 from 1st parity sows, 121 from 2nd to 4th and 46 from ≥5th) injected in the neck, by the intramuscular route, with 2 mL of saline as placebo.

In order to guarantee a totally homogenous management and the contact between vaccinated and unvaccinated animals throughout the study, pigs from the three groups were commingled since the moment of weaning. During the post-weaning phase, piglets were allocated in pens of approximately 45 animals; once in fattening, they were divided in pens of 15 pigs. This allocation was done by the farmers as usual, according to the animal weight. In both production phases, all the animals were allocated in the same building, in pens with totally slatted floor and completely solid walls, with feed and water ad libitum. The ventilation was regulated automatically depending on the farm interior temperature.

#### 2.2.1. Growth Performance and Mortality Index

The weight of each pig was measured at 25, 76 (coinciding with the end of the post-weaning phase) and 159 days old. The average daily weight gain (ADWG; g/pig/day) was calculated for the following periods: (1) between 25 and 76 days old, (2) between 76 and 159 days old and (3) the sum of both. Only the pigs with the three weight measures were included. The mortality index and the time of death were also registered.

#### 2.2.2. Serum Sampling

Serum samples were only collected in pigs from the first batch. In it, a stratified random sampling was performed when piglets were 26 days old, keeping the relation 1/2/1 in the number of sampled piglets according to 1st, 2nd to 4th and ≥5th sow parities, respectively. Thus, 40 animals from each vaccinated group and 20 from the control group were sampled and then identified with an extra tag. 

Serum samples were obtained at 26, 42, 63, 84, 105, 133 and 159 days old and were frozen at −80 °C until the analyses were performed.

#### 2.2.3. PCV2 Viremia

Serum samples from each sampling were pooled (5 pigs/pool), including in each pool only pigs from the same group and sow parity. DNA extraction was performed using the commercial kit High Pure PCR Template Preparation Kit, (Roche Diagnostics GmbH, Mannheim, Germany) following the manufacturer’s instructions. The isolated DNA was analyzed by qPCR using the commercial kit EXOone PCV2 oneMIX (EXOPOL S. L., Zaragoza, Spain). A sample was considered positive when Ct ≤ 42 for the PCV2 detection channel. Samples from positive pools were individually analyzed to quantify the PCV2 viral load. A synthetic DNA provided with the kit was used as the positive control and molecular grade water as the negative control. To quantify PCV2, standard curves were calculated by preparing serial ten-fold dilutions (5 × 10^5^–5 × 10^1^ copies/μL) of the DNA positive control. All qPCR reactions were run on an Applied Biosystems ABI Prism 7500 thermocycler (ThermoFisher Scientific, Waltham, MA, USA).

#### 2.2.4. PCV2 and *M. hyopneumoniae* Serology

All serum samples were tested for the presence of antibodies against PCV2 using the Alphalisa PCV type 2 (ALPHALISA PCV-orf2, in-house MSD AH, Boxmeer, The Netherlands). Only the serum samples taken at 26, 84, 105, 133 and 159 days old were analyzed to detect antibodies against *M. hyopneumoniae* using the ELISA M. hyo Ab Test (IDEXX Laboratories). Serum samples were considered positive to PCV2 IgG antibodies if the ELISA titer was higher than 4.3 Log2 according to the manufacturer’s instructions; for PCV2 seronegative samples, the titer was considered 4.0 Log2. For *M. hyopneumoniae*, the results were expressed as positive (S/P ratio higher than 0.4) or negative.

#### 2.2.5. Lung Evaluation

Lung evaluation was performed on pigs from both batches and always by the same researcher. In order to evaluate lesions compatible with *M. hyopneumoniae*, a pulmonary lesion index was calculated based on the scoring system established by Madec and Kobisch (1982) [17], taking into account the relative volume of each pulmonary lobe [20] and the presence of scars in any lobe [21]. Thus, the pulmonary lesion index was calculated as it follows: each pulmonary lobe was scored from 0 to 4 points according to the percentage of consolidated surface (0: no lesion; 1: <25% of the surface; 2: ≥25% to <50%; 3: ≥50% to <75%; 4: ≥75%), then each lobe score was multiplied by its corresponding relative volume (right cranial: 0.11; right middle: 0.10; right caudal: 0.34; left cranial: 0.05; left middle: 0.06; left caudal: 0.29; accessory: 0.05). The final score was calculated as the sum of all lobe scores and 1 extra point was added if any scar was present. 

An additional lung check was performed to evaluate lesions compatible with pleuropneumonia using the Slaughterhouse Pleurisy Evaluation System (SPES) method described in Dottori et al. (2008) [22]. Briefly, a score between 0 to 4 was given to each animal (0: absence of pleuritis lesions; 1: pleural adherence between the cranial lobes or at the ventral border of the lobes; 2: dorso-caudal monolateral focal lesion; 3: bilateral lesion of type 2 or extended monolateral lesion (at least 1/3 of one diaphragmatic lobe); 4: severely extended bilateral lesion (at least 1/3 of both diaphragmatic lobes)). This additional evaluation was done to determine whether the vaccination against *M. hyopneumoniae* has a secondary benefit in the reduction of *Actinobacillus pleuropneumoniae*-like lesions. Lungs with a score of 4 in SPES were not included in the analysis of *M. hyopneumoniae* compatible lesions (the multiple fibrinous adhesions to the costal pleura impeded the evaluation of mycoplasma-like lung lesions).

### 2.3. Statistical Analysis

Linear models were used to analyze the association of the continuous dependent variables: weight, ADGW and pulmonary lesion index with the vaccination group and sow parity. A linear regression analysis was also used to evaluate the differences in the PCV2 antibody titer between the three groups at each sampling age. A logistic regression was used to compare discrete variables (mortality rate, proportion of *M. hyopneumoniae* seropositive pigs and proportions of pigs with pulmonary consolidation, with pulmonary scars and with pleuritis) between groups, as well as to evaluate a possible interaction according to sow parity. Models combining both predictors and the interaction between them were evaluated and the final model was selected using the likelihood ratio test and the Aikake’s information criterion (AIC). 

The Friedman test of repeated measures and the McNemar test were used to detect the PCV2 seroconversion and the *M. hyopneumoniae* seropositivity increase in each group. These were considered when a significant increase in the PCV2 antibody titer or in the *M. hyopneumoniae* seropositive proportion between two consecutive samplings, respectively, was observed. For these analyses, only the results from the pigs which were available in all the samplings (a total of 34 from Group A, 32 from B and 18 from C) were employed. *p* values were adjusted using the Bonferroni correction in both cases.

Data analysis was performed in R v. 4.0.3 (Vienna, Austria) [23]. In all analyses, a *p <* 0.05 was considered as significant.

## 3. Results

### 3.1. Mortality Rate

Global mortality rates in each group are shown in Table 1. The mortality rates in the vaccinated groups (A and B) were always lower than in the control group (2.35 and 2.90% vs. 3.54% during nursery; 3.85 and 2.99% vs. 5.05% during fattening respectively). The Odds Ratio (OR)of the global mortality rates and 95% Confidence Interval (CI) from each vaccinated group regarding the control one are shown in Table 2. An association between sow parity and mortality was not found (*p* > 0.05), although the overall mortality in pigs from primiparous sows was slightly higher (8.05%) than in the other ones (6.47% and 6.06% in 2nd to 4th and ≥ 5th respectively).

### 3.2. Pig Weight and Growth Performance

The weight of the pigs and the ADWG could be assessed in 583 out of 602 animals. The remaining ones were excluded due to ear tag losses or deaths. 

The results of the weight of the pigs at each age and the ADWG for each production period are detailed in Table 1. Significant differences in the weight were only detected between animals in Group A and Group C, as those from Group A showed a significantly higher final weight (*p =* 0.014). Regarding the ADWG, a significant increase was detected in the 76–159 days period in Group A compared to Group C (*p =* 0.006). No differences were detected in the rest of the paired comparisons. The overall growth performance (25–159 days) was better in both vaccinated groups than in the unvaccinated one, but the difference was only significant in Group A (*p =* 0.010). The final model did not include an interaction between vaccination groups and sow parity (*p* > 0.05).

### 3.3. PCV2 Viremia

A total of 675 serum samples were analyzed to detect PCV2 DNA: 267 from Group A, 276 from Group B and 132 from Group C. Twenty-five serum samples were not available due to the impossibility to identify the corresponding animal in some samplings or its death. 

**Figure 1:**
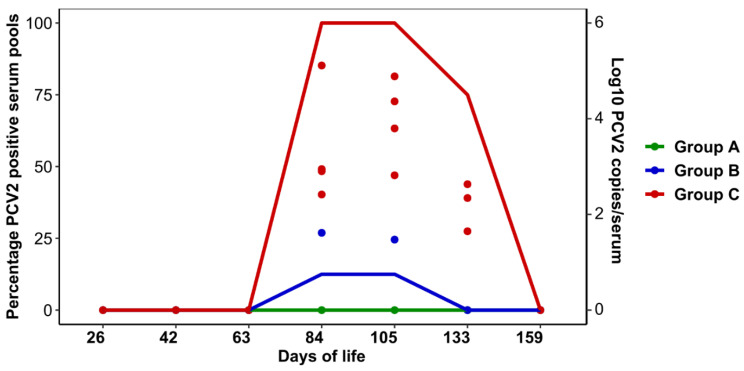
Dynamics of PCV2 viremia in each group. Lines represent the percentage of PCV2 positive serum pools (5 pigs/pool) at each age. Points represent the number of PCV2 copies/mL of serum in positive pools.

The results of PCV2 viremia are shown in Figure 1. PCV2 was never detected in serum samples from Group A and was only found in two pools from Group B: one corresponded to pigs from 2nd to 4th parity sows and the other to animals from 1st parity sows (two and three individual samples respectively; viremia ranged from 1.72 × 10^2^ to 1.76 × 10^3^ copies/mL). In contrast, in Group C, the infection was detected from 84 to 133 days old and the viremia ranged from 2.60 × 10^2^ to 1.29 × 10^5^ PCV2 copies/mL serum.

### 3.4. Serologic Response to PCV2

**Figure 2:**
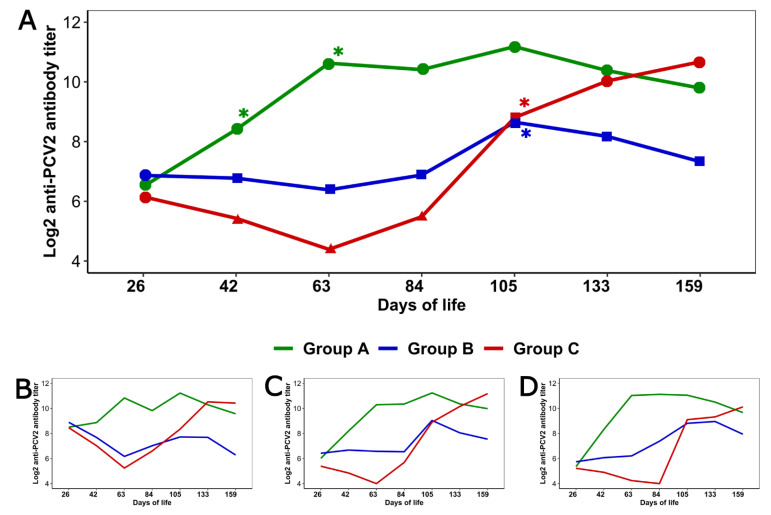
Dynamics of PCV2 serology. (**A**) shows the evolution of PCV2 serology in each group. Different shapes indicate significant differences in the anti-PCV2 antibody levels between the three groups at each sampling. The asterisk (*) indicates the age at which seroconversion was detected in each group. (**B**–**D**) represent the dynamics of PCV2 serology in each group for pigs from 1st parity sows (**B**), 2nd to 4th parity sows (**C**) and ≥ 5th parity sows (**D**).

PCV2 serology differed between the three groups (Figure 2A). Vaccinated groups presented seroconversion in different moments: from 26 to 42 and from 42 to 63 days old (*p <* 0.001 for both intervals) in Group A; and from 84 to 105 days old (*p =* 0.037) in Group B, coinciding with the detection of PCV2 circulation in this latter case. In the unvaccinated group, seroconversion occurred between 84 and 105 days old (*p =* 0.01). 

The levels of anti-PCV2 antibodies were only similar for the three groups at 26 days of age (*p* > 0.05). Figure 2A shows the significant differences among the three groups at each age.

Figure 2B–D show the anti-PCV2 antibody dynamics of both vaccinated groups and the control one in the pigs from each of the mentioned sow parities. The final model did not include an interaction between vaccination groups and sow parity (*p* > 0.05); thus, the three dynamics were similar. However, two observations should be mentioned: in all groups, at 26 days old the anti-PCV2 antibody titers were significantly higher in piglets from primiparous sows than in those from multiparous sows (*p <* 0.05); in Group B the antibody levels of pigs from primiparous sows showed a continuous decrease from 26 to 63 days old, in contrast with what was observed in those from the other parities.

### 3.5. Serologic Response to M. hyopneumoniae

**Figure 3:**
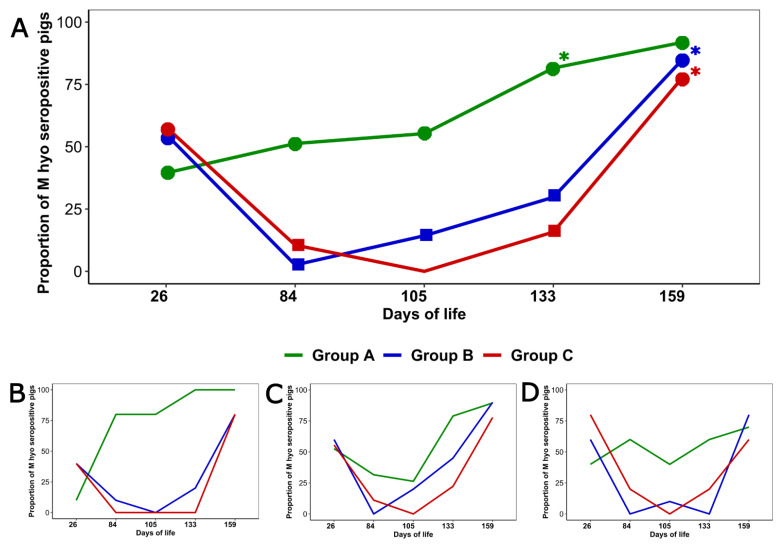
Dynamics of *M. hyopneumoniae* seropositivity. (**A**) shows the percentage of seropositive pigs to *M. hyopneumoniae* in each group. The different shapes indicate the significant differences in the percentage of *M. hyopneumoniae* seropositivity between the three groups at each sampling (at 105 days old all the piglets from Group C were seronegative). The asterisk (*) indicates the age at which a significant increase in the proportion of *M. hyopneumoniae* seropositivity was detected in each group. (**B**–**D**) represent the *M. hyopneumoniae* seropositivity in each group for pigs from 1st parity sows (**B**), 2nd to 4th parity sows (**C**) and ≥ 5th parity sows (**D**).

At 26 days old the proportion of *M. hyopneumoniae* seropositive piglets did not differ significantly among the three groups (*p* > 0.05); afterwards, this proportion was always higher in Group A than in Groups B and C (*p <* 0.05) (at 105 days old all the piglets from Group C were seronegative), with the only exception of the last sampling (Figure 3A). The results of the logistic regression using Group C as the reference are shown in Table 2. The age at which a significant increase in the proportion of seropositive pigs occurred in each group is indicated in the Figure 3A.

Figure 3B–D show the evolution over time of the proportion of *M. hyopneumoniae* seropositive pigs in each group by sow parity. Sow parity was not found to be associated with the proportion of *M. hyopneumoniae* seropositivity (*p* > 0.05); however, at 26 days old the proportion of *M. hyopneumoniae* seropositive piglets was lower among pigs from primiparous sows than among those from the other parities (*p <* 0.05).

### 3.6. Evaluation of Lung Lesions

Lung check was possible in 269 pigs (44.68% of the pigs which finished the study), resulting in the evaluation of 260 lungs for the presence of mycoplasma-like lung lesions: 87, 89 and 84 from groups A, B and C, respectively. The remaining nine showed a Grade 4 in the SPES scale and were excluded for this evaluation. The results of the lung check are shown in Table 3. The pulmonary index was better in both vaccinated groups than in the unvaccinated one, but this difference was only significant in Group A (*p =* 0.018). 

Table 2 shows the results of the comparison of groups A and B using the Group C as the reference. The final model did not include an interaction between vaccination/placebo groups and sow parity regarding neither pulmonary index, pulmonary consolidation nor scar presence (*p* > 0.05).

One hundred and seventy-five out of 269 evaluated pigs (65.05%) showed lesions compatible with pleuropneumonia according to the SPES (Table 3). The lowest percentage of pigs with pleuritic lesions was observed in Group B, although without a significant statistical difference regarding the other groups (*p* > 0.05). The results of the comparison of the vaccinated groups regarding the control one are shown in Table 2. Sow parity was not found to be associated with the proportion of pigs with pleuritis (*p* > 0.05).

## 4. Discussion

In this study, the efficacy of two commercial ready-to-use PCV2 and *M. hyopneumoniae* vaccines, regarding an unvaccinated group, was assessed under field conditions in a herd infected by PCV2 which had a history of enzootic pneumonia. In addition, sow parity was considered as a variable which could influence some of the obtained results, a situation that, to our knowledge, has not usually been considered in comparative studies under field conditions.

The protection provided by the PCV2–*M. hyopneumoniae* ready-to-use vaccines seemed to show an improvement of the ADWG and the mortality rates at herd level. Although the difference in the mortality rate was not significant, we observed a slight improvement in both vaccinated groups for this parameter. In the case of the ADWG, as in other comparatives studies of bivalent PCV2–*M. hyopneumoniae* vaccines, no variation between the three groups was found over the nursery period, although differences emerged during the fattening phase; this was to be expected, as both infections occurred at this stage [24,25]. Using the unvaccinated group as a reference, the improvement in the ADWG was only significant in Group A. This may be directly related to the significant reduction in the pulmonary lesion index and the absence of PCV2 viremia detected in this group during the study. In group B, although an improvement in the ADWG was observed regarding the control group, the higher pulmonary lesion index and the emergence of PCV2 infection in a reduced number of pigs may have resulted in the non-significance of the improvement of this parameter.

Our results are consistent with previous field studies that used these or other PCV2-*M. hyopneumoniae* bivalent vaccines [24,26], showing that bivalent vaccination reduces the number of infected animals and the viremia. However, in this study two points should be highlighted. First, the PCV2 load in the unvaccinated group was lower than the observed in pre-trial analysis. Regarding this, we hypothesize that housing together vaccinated and unvaccinated pigs reduced the number of susceptible animals. Since fewer susceptible animals imply fewer pigs that get infected, and therefore fewer shedder pigs and lower PCV2 exposure, this particular management may have contributed to a lower viremia in infected animals [27,28,29]. Second, when the peak of PCV2 infection in the control group occurred, PCV2 viremia was only detected in a few cases from Group B and was not detected in Group A. These results indicate that both vaccines provided a high protection when pigs were exposed to a source of PCV2. Moreover, it must be mentioned that, under real field conditions, in which all pigs would be vaccinated, the effectiveness of both vaccines, measured as the detection of PCV2 viremia, should be similar or even better than the observed in this study. 

In the moment of vaccination all piglets tested negative to PCV2 qPCR, so the antibodies detected at that age were of maternal origin. However, despite the fact that all animals started from an equal situation, PCV2 serological dynamics differed among the three groups. The unvaccinated group showed the typical evolution, with a first decrease in anti-PCV2 MDA and the consequent increase following the infection [30]. Each vaccine showed its own dynamics. In pigs from Group A, the vaccine induced PCV2 seroconversion shortly after its administration. This was not observed in those from Group B, in which the seroconversion occurred later, coinciding in time with the infection in some animals from this group and from the control one. Both observations are in line with previous information regarding each vaccine [31,32]. The different composition of the vaccines may have influenced the obtained results: while the vaccine used in Group A consists in PCV2 ORF2 expressed in a baculovirus system [31], the one used in Group B is a chimeric PCV1 virus expressing PCV2 ORF2 [32]. In addition, the vaccine adjuvant is also different, which has also been suggested to influence the PCV2 immune response [33]. Thus, according to the obtained results, the rapid seroconversion and the consequent higher levels of anti-PCV2 antibodies in Group A could be one of the reasons which would explain why no infected pigs were detected in this group. 

A different pattern in the PCV2 immunological response following vaccine/placebo administration was observed in the considered sow parities. However, the final model did not show a significant interaction effect between these two variables. That possibility had to be evaluated because, in this particular herd, we detected higher anti-PCV2 antibody levels in pigs from primiparous sows than in those from the other parities, as it was to be expected. In situations like that some immunological effects of the vaccines could be masked by a situation of high anti-PCV2 antibody levels in gilts and high MDA levels in their piglets [12,34]. In relation to this, in Group B, different PCV2 serological dynamics were observed in the piglets from primiparous sows than in those from the multiparous ones, in which the vaccination avoided the anti-PCV2 MDA declination; however, in those from primiparous sows the evolution was quite similar to that of unvaccinated pigs. This observation prevents us from totally ruling out a possible interference between the used vaccine and high levels of MDA.

In situations when both gilts and sows are unvaccinated against PCV2, as in this study, some of them could have been previously infected and transmitted the infection to their offspring [35,36]. The latter did not occur in this study, as we did not detect infected piglets in the first sampling; however, the higher MDA in piglets from primiparous sows suggests a recent PCV2 infection of them, probably before mating or even during their gestation [36]. Nevertheless, in recent years the immune situation of the breeding collective is changing because vaccinating sows against PCV2 has become a common practice. Subsequently, in this new context, the PCV2 MDA levels in the offspring tend to increase with each parity [8,13]. Therefore, a regular surveillance of the PCV2 MDA levels would be useful to minimize the risk of a possible interference between the MDA and the ready-to-use vaccines and, thus, achieve the maximum potential of these products.

Regarding the *M. hyopneumoniae* serology, our results differ from previous information. In the case of Group A, the significant increase in the proportion of seropositive pigs did not seem to occur immediately after vaccination, unlike in previously published data [31]. In the case of Group B, after vaccination, we observed a decrease in the proportion of seropositive pigs which was not reverted until 84 days old, when it started to increase. This observation is contrary to the previous results obtained in field conditions [26]. A possible reason for both differences may be that, at the beginning of our study, some of the included piglets were already seropositive, unlike in the aforementioned studies which were performed on seronegative animals. However, this should have been confirmed by analyzing the serum samples from days 42 and 63. Furthermore, a lower proportion of seropositive animals was observed among the piglets from primiparous sows than among those from higher parities, which is coherent with previous information [37].

Previous studies have shown a significant reduction of lung lesions compatible with enzootic pneumonia with the administration of any of the two ready-to-use vaccines [25,26]. In this case, although both vaccines reduced the affected lung tissue, this decrease was only significant in Group A. This observation might be related to the different *M. hyopneumoniae* strains included in each vaccine and their degree of similarity to the field strain, to the vaccine adjuvant or even to the protection conferred by seropositivity; the latter might also be the reason why the group with higher seropositivity rate showed a lower percentage of pigs with pulmonary consolidation. With regard to *Actinobacillus pleuropneumoniae*-like lesions, no difference was observed between groups. Moreover, no variations neither in the enzootic pneumonia lesions nor in the pleuropneumonia ones were observed regarding the sow parity.

## 5. Conclusions

The two ready-to-use PCV2 and *M. hyopneumoniae* vaccines seem to improve all the measured parameters in a herd affected by these infections under field conditions. However, only the ADWG and the index of pulmonary lesions compatible with enzootic pneumonia in Group A were significantly better than those observed in the control group. Besides, this field study shows that the sow parity did not influence the vaccine outcomes, although the anti-PCV2 antibody response might be slightly different in pigs from primiparous sows than in those from other sow parities depending on the employed vaccine.

## Figures and Tables

**Table 1:** Mortality rate and mean ± standard deviation (sd) of the pig weight and average daily weight gain (ADWG) for each group.

Group (*n* of Pigs Used for Calculating the Mean and the sd/Total Starters)	Mortality Rate (Dead Pigs from Total Starters)	Weight at Inclusion (25 Days Old) (kg)	Weight at 76 Days Old (kg)	Final Weight (159 Days) (kg)	ADWG 25–76 Days (g/day)	ADWG 76–159 Days (g/day)	Global ADWG (25–159 Days) (g/day)
Group A (196/213)	6.10% (13) ^a^	6.81 ± 1.46 ^a^	26.33 ± 4.90 ^a^	95.77 ± 12.67 ^a^	373 ± 77 ^a^	861 ± 116 ^a^	670 ± 86 ^a^
Group B (186/207)	5.80% (12) ^a^	6.68 ± 1.22 ^a^	26.62 ± 4.95 ^a^	94.00 ± 12.64 ^a, b^	383 ± 84 ^a^	835 ± 118 ^a, b^	658 ± 89 ^a, b^
Group C (201/226)	8.41% (19) ^a^	6.52 ± 1.25 ^a^	25.72 ± 4.65 ^a^	92.23 ± 12.87 ^b^	369 ± 78 ^a^	827 ± 128 ^b^	647 ± 92 ^b^

^a, b^: Different letters indicate significant differences among groups.

**Table 2:** Results of the logistic regression using the control group (Group C) as the reference.

Variable		Group A				Group B			
		Estimate	*p* Value	OR	95% CI	Estimate	*p* Value	OR	95% CI
**Mortality Rate**		−0.348	0.351	0.706	0.332–1.456	−0.404	0.291	0.667	0.307–1.396
Proportion of *M. hyopneumoniae* seropositive pigs	26 days old	−0.799	0.178	−0.449	0.135–1.418	−0.145	0.804	0.864	0.267–2.707
	84 days old	2.442	0.005	11.501	2.553–85.82	−1.521	0.233	0.218	0.009–2.503
	105 days old *	18.397	0.990	9.77 × 10^7^	2.21 × 10^−29^- NA	16.626	0.991	1.66 × 10^7^	2.89 × 10^−29^ - NA
	133 days old	3.570	<0.001	35.515	7.93–221.76	0.843	0.259	2.323	0.583–11.83
	159 days old	1.171	0.165	3.224	0.614–18.78	0.458	0.535	1.581	0.346–6.679
Proportion of pigs with lobe consolidation		−0.571	0.148	0.564	0.255–1.211	−0.188	0.649	0.829	0.364–1.858
Proportion of pigs with scars		−0.002	0.995	0.998	0.545–1.826	0.043	0.888	0.957	0.524–1.751
Proportion of pigs with pleuritis		−0.262	0.418	0.770	0.408–1.446	−0.464	0.148	0.629	0.333–1.173

* All tested pigs from control group were seronegative to *M. hyopneumoniae*. NA: Not available.

**Table 3:** Results of the evaluation of lung lesions.

Group	Lesions Compatible with *M. hyopneumoniae*			Slaughterhouse Pleurisy Evaluation System (% Pigs)				
	Lobe Consolidation (% Pigs)	Presence of Scars (% Pigs)	Pulmonary Lesion Index (Mean ± s.d.)	Grade 0 (No Pleuritis)	Grade 1	Grade 2	Grade 3	Grade 4
Group A	75.86 ^a^	50.57 ^a^	0.96 ± 0.63 ^a^	35.16 ^a^	36.26	14.29	9.89	4.40
Group B	82.02 ^a^	49.44 ^a^	1.10 ± 0.61 ^a,b^	40.00 ^a^	48.89	6.67	3.33	1.11
Group C	84.52 ^a^	51.19 ^a^	1.18 ± 0.6 ^b^	29.55 ^a^	45.45	13.64	6.81	4.55

^a, b^: Different letters indicate significant differences among groups. s.d.: standard deviation.

## Data Availability

The data are available from the corresponding author upon reasonable request.
